# Book Review: Stress Less, Accomplish More: Meditation for Extraordinary Performance

**DOI:** 10.3389/fpsyg.2020.01830

**Published:** 2020-08-07

**Authors:** Surabhi Lodha, Rashmi Gupta

**Affiliations:** Cognitive and Behavioral Neuroscience Laboratory, Department of Humanities and Social Sciences, Indian Institute of Technology Bombay, Mumbai, India

**Keywords:** meditation, stress, manifesting, mindfulness, Ziva meditation

While browsing through the Internet, the authors tried to find a brief meditation technique to practice during the COVID-19 pandemic. They came across the videos of Emily Fletcher teaching Ziva meditation online. It drove them to her book titled “Stress less, accomplish more.”

Emily Fletcher is the founder of Ziva Meditation and the creator of zivaONLINE, the world's first online meditation training program. A powerful meditation practice transitioned her from being a young but anxious and insomniac Broadway actress to a dedicated practitioner and a flourishing meditation professional. In this book, she introduces a new technique called “Z-technique,” adapted from her live, in-person Ziva meditation. This technique is specially designed for high-performing, outcome-driven individuals, teaching them to be less stressed while increasing productivity and achieving success.

The first chapter points out that people often cannot meditate because of super-busy schedules, fear of failure (the meditation shame spiral), or vagueness about the practice. It educates on how and how not to approach meditation. While introducing the Z-technique, the author argues that investing only 25 min twice a day to this technique could significantly improve the remaining hours qualitatively.

Chapters two and three explain that meditation gives us the control to regulate our response to perceived stress. The long-term effect of stress from work, family, relationships, etc., is toxic and debilitating, manifested in decreased work performance, reduced attention span, irritability, mood swings, etc. The concept of adaptation energy is reintroduced here (Gorban et al., 2016) as a psychological measure of one's stress-coping capacity. This reservoir could be detoxified from accumulated stress and abundantly replenished with energy by de-exciting the nervous system through meditation.

The fourth chapter further argues that this Internet era, which equates rest or relaxation with laziness and stagnancy, has seen a spike in sleep-related problems like insomnia and restlessness. The author asserts that both sleep and meditation are equally crucial for a healthy life. While sleep cleans our brain by cleaning out toxic by-products (Xie et al., 2013), meditation rests our entire body, including the brain.

Adding to the further benefits of mediation, in the fifth and the sixth chapters, it is suggested that meditation revamps the body by eliminating the built-up stress in the form of disturbed sleep, organ inflammation, chronic acidity, dullness, and pain. Moreover, regular meditation practice improves immune functions and treats disorders like burnout and chronic fatigue, depression, anxiety, infertility, Irritable Bowel Syndrome, migraines, Parkinson's disease, pain, etc. It improves longevity and quality of life combined with Ayurveda—proper diet, exercise and yoga practices, and environmental harmony. The above claims are supported by providing neurological evidence like changes in the right and left-brain, amygdala, insula, corpus callosum, and telomere.

The seventh chapter posits that, unfortunately, contemporary culture is built on the “I'll be happy when syndrome,” which is an abstract idea that one's happiness is based on future achievements. This far-fetched pursuit is exhausting and bewildering. But luckily, meditation helps us pull out of the future, settles into the present, and instills the fact that bliss and contentment are within us, independent of external situations.

The explanation of the harmful effects of stress and how meditation eradicates them is followed by the description of Ziva or the Z-technique in chapter eight. Ziva is born out of Nishkam Karma Yoga of Indian spiritual traditions, which requires no focused or effortful concentration or a forcible mind clearing (Diwan and Kamra, 2018). The Z-technique is a sequenced combination of three “Ms”: Mindfulness, Meditation, and Manifesting. This simple 25-min technique begins with mindfulness of 2–3 min (aware and completely present), similar to the “open awareness meditation” style (Lutz et al., 2008). It is followed by 14–16 min of meditation (healing from the past), which includes the sub-vocalization of an impersonal word like “one.” The author calls this “whisper of an echo,” which lets our body and mind drift into a deeply relaxed state spontaneously.

The technique ends with 2–3 min of manifesting (consciously creating and planning our future). One offers gratitude to everything, accompanied by seeing dreams as unfolding in the present, and not merely magical thinking.

The ninth and tenth chapters further explain that the Z-technique enables us to detect the subtleties and patterns in our daily lives as it expands consciousness, making us more attuned to the sensations, thoughts, and feelings. Hence, we become more intuitive and insightful and able to enter the “flow state.” This unveiling of full potential is termed as “up-leveling,” marked by extraordinary performance and fulfillment.

The subsequent chapters explore the prospect of meditation as a tool for the development of collective consciousness. If one individual consciously learns to break the old habits by finding a gap between the trigger and impulsive reactions, this transformation in consciousness permeates other beings. Keeping these in mind, we must make the Z-technique a non-negotiable daily practice. The author claims that, ultimately, this helps us become good in every sphere of life.

For the contemporary fast-paced and stress-ridden society, the book serves as a foundational and practical guide for people who want to improve their physical and mental well-being but don't know where to begin. It is an amalgamation of ancient meditation practices, modern neuroscience, and pop psychology sans metaphysics or spirituality. It makes meditation accessible and understandable to all, not just high-achievers. The repetitive explanation of stress and lack of empirically investigated data to validate the technique is a bit unsettling. However, the book is simple, refreshing, and rewarding.

In conclusion, the book addresses how meditation could remarkably improve productivity and efficiency in an overly stressed modern world. It emphasizes that meditation is much more than the austerity of a Himalayan Yogi. In meditation research, the issues of universality and secularity of a technique are not thoroughly addressed. Such streamlined meditation practice could be easily adapted by anyone who has struggled with commencement, commitment, and consistency. The current need is to give equal emphasis to study such techniques of “meditation for the ordinary.”

## Author Contributions

All authors listed have made a substantial, direct and intellectual contribution to the work, and approved it for publication.

## Conflict of Interest

The authors declare that the research was conducted in the absence of any commercial or financial relationships that could be construed as a potential conflict of interest.
